# Book Reviews

**Published:** 1822-07

**Authors:** 


### CRITICAL ANALYSIS

OF

### ENGLISH AND FOREIGN LITERATURE

RELATIVE TO THE VARIOUS BRANCHES OF

### Medical Science.

Quæ laudanda forent, et quæ culpanda, vicissim

Illa, prius, cretâ; mox hæc, carbone, *notamus.*—PERSIUS.

### DIVISION I.

### ENGLISH.

ART. I.—*Observations on those Diseases of Females which are attended by Discharges. Illustrated by Copper-plates of the Diseases.* By CHARLES MANSFIELD CLARKE, M.R.C.S. Lond. Part II. Royal 8vo. pp. vi. 243; four Plates. Longman and Co. London, 1821.

[Concluded from vol. xlvii. page 62.]

THE next subject which remains to be considered is that species of inflammation of the mucous membrane of the uterus which terminates in the secretion of pus.

In forming our diagnosis of the inflammatory diseases of the female organs of generation, it will be proper to notice that the secretions resulting from inflammatory action vary according to the tissue affected: some membranes secreting pus, some mucus, some a mixture of these, and others coagulable lymph. It may be remarked, also, that, when pus proceeds from an ulcerated surface, it is liable to be mixed with blood.

It is the property of some membranes to secrete both pus and coagulable lymph, according to the nature of the inflammation which affects them. The uterus, under painful menstruation, arising from inflammation of the mucous surfaces, discharges flakes of coagulable lymph, sometimes forming a cast of its cavity. But the mucous membrane of the uterus will also secrete pus, which may be discharged from the vagina as fast as secreted, or may be retained within its cavity by adhesive inflammation closing the os tincæ, or by the glands secreting a tough mucus filling up its cavity, or by a great contraction of the cervix itself. The author adds, that the vagina *rarely* gives rise to the production of layers of coagulable lymph, but very slight inflammation will excite purulent secretion. Pus, also, may be secreted by the inner membrane of the bladder, or meatus urinarius.

When the pus secreted by the uterus makes its way readily out of the vagina, it produces little besides heat and uneasiness in this passage, and may be mistaken for disease of the latter; but, when the pus is retained in the cavity of the womb, it produces *constant and severe pain in the back.* The womb, on examination, is tender to the touch, and its bulk is found increased in proportion to the quantity of the contained matter. Sometimes a sudden burst takes place, and pus of a very offensive odour escapes, which throws light on the nature of the previous enlargement.

It is curious that, notwithstanding the preceding description (which is abridged from the author) speaks of constant and severe pain in the back as a symptom, without any qualification, the first case related, which terminated fatally, ran its course "unattended by any pain." These are the particulars:—For a few weeks the patient had a small discharge, at first like fluor albus, which afterwards became of a brownish colour, offensive to the smell, and greater in quantity. On examination, the os uteri was found ragged, and harder than usual. From the upper part of the cervix a tumor bulged out in all directions, so as to occupy nearly the whole space from the pubis to the sacrum. Towards the end of the month, she was suddenly seized with violent pain in the lower part of the abdomen, with a sensation as if something had suddenly given way; she grew faint, having a thread-like pulse; the extremities became cold, and in the evening she died.

Here no pain existed in the back; that in the abdomen arose from the effusion of a quantity of acrid pus into its cavity, by the bursting of the uterus. On dissection, seven or eight ounces of a most offensive fluid were found in the abdomen. The intestines were, of course, inflamed by the presence of this matter. The uterus was burst, and in a gangrenous state, containing about five ounces more of pus. Its internal surface had a honey-comb appearance, with something like coagulable lymph. The orifice between the cavity and the cervix uteri was closely contracted.

Another case is related, in illustration of the nature of this complaint. Inflammatory action ran high, and the uterus was discovered to be distended, on examination. Hectic fever had supervened; when, at the end of several weeks, a sudden discharge of pus issued from the vagina, and the patient recovered.

When the uterus has acquired the bulk which it usually possesses at the fourth month of pregnancy, it may be distinguished from carcinoma, which the author has never seen of that size. It may be distinguished from the fleshy tubercle, from the rapidity with which the collection of pus takes place, in comparison with the growth of the other.

The treatment, during the presence of inflammatory symptoms, should be local or general bleeding, with hip bath, purging, opiates, &c. When the uterus is distended with pus, it will be proper to introduce, through its orifice, a male catheter, for the purpose of evacuating its contents.

*Inflammation of the mucous membrane of the vagina* may arise from the same circumstances which produce inflammation in other parts,—even from ordinary connexion between the sexes. It is also frequently produced by the venereal affection, and is then termed gonorrhœa. The treatment, in either case, is too well known to require particular notice here.

*Abscess of the Vagina* is of two kinds: the one is seated in the labia or nymphæ; the other in the cellular membrane surrounding the vagina. The latter alone occupies the attention of the author. When an abscess forms in the cellular membrane of the vagina, the discharge is not constant, as from an ulcerated surface; the patient, after the evacuation of a quantity of matter, is free from discharge for a time. The disease is very untractable; as, when it bursts, the opening of the abscess rarely takes place in the depending part, being generally high up, beyond the reach of the remedies.

Mr. Clarke relates three cases, two of which terminated favourably under the use of constitutional treatment. In one of them,

"On examination, a bag distended with fluid could be felt behind the vagina, and, the lower part of this being pressed upon, a highly offensive purulent discharge, of a greenish colour, came from the upper part of the vagina. The pressure being continued, all the matter escaped, and the bag could be no longer felt. Thus it was satisfactorily explained why the symptoms were diminished by the coition, and how this produced an evacuation of the contents of the bag.

"It was not judged proper to resort to any chirurgical operation: a plan of treatment was directed, having for its intention the improvement of the health of the patient, and the prevention of accumulation in the cavity of the abscess.

"The patient returned into the country; the powers of her constitution were restored; the discharge diminished, and ceased to be offensive; pregnancy took place, and the patient was delivered prematurely, in consequence of some exertion which she had undergone."

The treatment of the third case was not so successful: it was that of "a young lady, who was attacked with pain about the pelvis, attended with soreness of the vagina, and she was incapable of bearing the slightest exercise in a carriage without an aggravation of all the symptoms. A brownish puriform discharge, of an offensive smell, was occasionally voided; the functions of the stomach were disturbed; the powers of the constitution flagged, as well as those of the mind. An examination was made by the author; but he discovered little, except that, on the removal of the finger, a purulent discharge followed. The uterus was perfectly healthy."

*Ulceration of the Os and Cervix Uteri* is of two kinds, the *corroding* and *carcinomatous ulcer*, which generally take place at the cessation of the catamenia, when diseases of increased action in the parts of generation may be expected. The author remarks, that,

"When once ulceration has produced a breach of surface in a membrane, it frequently happens that such ulceration extends itself over continuous parts of such membrane, so as to enlarge its surface: in other instances, the process involves the more deep-seated parts, causing an excavation, with no enlargement of the original boundary of the ulcer.

"In the corroding ulcer of the os uteri, the membrane which covers it first takes on disease, and very shortly afterwards the ulcer extends to the whole circumference of the opening, and to the parts immediately beneath it; so that the natural shape of the os uteri is destroyed. Thence the ulceration proceeds to the cervix, and consumes it; so that, if the patient should die in this stage of the disease, nothing will be found, after death, but the body and the fundus of the uterus. Sometimes the disease does not stop here, but, before the patient is destroyed, the absorbents employed in the process of ulceration will have taken up nearly the whole body of the uterus, so that very little more than the fundus will remain."

In the carcinomatous ulcer, the same destruction does not take place. On examination of the corroding ulcer, the breach of surface may be readily distinguished, but no hardness or thickness of the parts, from deposit of new matter, as in cancer. The author considers that it is peculiar to cancerous inflammation to deposit new matter by the arteries, which more than counterbalances the effect produced by the action of the absorbents, and the thickening and destructive process go on simultaneously.

Carcinomatous tumor of the cervix uteri, and of the rectum, were treated by the author in his former volume: he now confines his attention to the ulcerative stage of the same disorders. The fatality of the uterine affection will be, *cæteris paribus*, accelerated in a ratio with the extent of the ulcerated surface. The ulcerative process is more rapid in its commencement than afterwards, which the author ascribes to the failing powers of the patient; a fact which the practitioner should keep in view, for the purpose of regulating his prognosis. Debility, whether the natural consequence of the disease or of hemorrhages from the ulcer, will retard its progress.

*Ulcerated Carcinoma of the Rectum.*—The vicinity between the rectum and the uterus, and the sympathy between them, produce sometimes confusion in the diagnosis of these disorders: complaints of the rectum having been treated as diseases of the uterus, and *vice versa.* The early stage of carcinoma of the rectum is attended with a mucous discharge, which becomes gradually purulent, and its quantity increases according to the length of the diseased portion of gut. The presence of pus may induce a suspicion of fistulæ; but, on examination by the finger, the rectum will be found girt, and its passage impeded, by a constriction of considerable thickness; and any attempt to surmount the difficulty by violence will produce excruciating pain and discharge of blood. The surface of the constricted part will be found abraded.

As the fatality of this disease depends more upon the obstruction to the passage of the feces through the intestine, than to the symptoms peculiar to cancer, the same consequence will result from a carcinomatous thickening of the gut to a small extent, as from a large portion being involved in the disease; hence the surgeon need not be over anxious about discovering its extent. Thus, as the author remarks, "carcinoma, affecting not more than a quarter of an inch of the rectum, may, by obstructing the passage of the feces, cause a distention of the whole colon; and the patient may die of inflammation of the coats of the gut produced by such distention. If three inches of the intestine had been involved in the same disease, the symptoms would only have been the same; so that, neither will the treatment be improved, nor the prognostic be assisted, by the knowledge of the extent of the complaint."

"All the symptoms which attend the first stage of this disease will be found to exist in a greater degree in the second. The darting pain will be increased both in frequency and in violence; the action of the heart will be greatly and permanently accelerated; the functions of the stomach will become more and more impaired; vomiting will be almost constantly present; temporary relief will be found only in opium; and permanent rest only in the grave. In the progress of the ulceration, blood-vessels will be exposed, which will pour out, according to their size, a larger or a smaller quantity of blood; and happy would it be for the patient if such hemorrhage should prove fatal; but such an event is hardly to be expected, and, unless in parts more abundantly supplied with blood than the rectum, such an occurrence is seldom met with.

"The ravages of carcinoma extend in all directions; most where the disease is least resisted. Thus it will occasionally happen that the parts which form the barrier between the rectum and the vagina will be destroyed, and a communication will be formed between the two cavities; or it may happen that distention of the upper part of the rectum by feces above the seat of the disease, may cause common ulceration of the coats of the intestine and of the vagina; and the feces may, from this cause, also be voided through the latter passage during the continuance of the patient's life. From this point of time, the disease in the rectum proceeds after the manner of external carcinoma; the part in which it exists, having ceased to perform its accustomed functions, becomes no longer annoyed by serving the purpose of a canal."

To the symptoms which accompany carcinomatous ulceration when it is confined to the uterus, and its surrounding cellular membrane, will be superadded others, when a new organ is attacked.

"For instance, when the rectum is attacked, there is tenesmus, great heat in that part, increased distress in voiding the feces, exquisite tenderness of the gut if the finger be carried into it. So, in like manner, if the disease proceeds to the bladder, shivering usually comes on, succeeded by heat, great pain, which is fixed and constant, together with strangury. If the disease makes its way, which however is not very common, into the cavity of the abdomen, symptoms of peritoneal inflammation will present themselves; such as tenderness of the belly, distention of its cavity, and a small frequent pulse. Now, if these symptoms are allowed to proceed, the patient will die, as she would do if attacked by acute inflammation of the bladder, or of the peritoneum. Thus the presence of these symptoms, even in a late period of the disease, may call for the loss of blood, although, under any other circumstances, it would be improper to direct such a remedy."

Among the symptoms of corroding ulcer, we may notice a sensation of heat in the region of the uterus. Instead of the menstrual secretion, a yellowish discharge escapes, perhaps trifling, mixed with blood. The sensation of heat, and quantity of discharge, increase as the ulceration extends. The countenance becomes pallid, and weakness of the whole system takes place. Pain, to a certain extent, may be felt; but there is no lancinating pain, as in the carcinomatous ulceration; and, when the finger is pressed on the ulcerated part, the patient does not suddenly shrink, as in cancer, but complains of a sense of soreness. The corroding ulcer, although distinct from cancer, is no less uniformly fatal; yet will last a greater length of time, unless it be attended by violent hemorrhages, which arise from the exposure and ulceration of blood-vessels.

In describing the symptoms of carcinomatous ulceration, the author remarks, that,

"At the commencement of the ulcerative process, it is not unusual for the patient to complain of a puffy and enlarged state of the external organs, which owes its origin simply to the increased action and distention of the vessels in the neighbourhood. A great degree of itching is another frequent attendant, and erysipelas occasionally takes place in the vicinity.

"The cuticle is often found to desquamate; a trifling oozing ensues, which dries upon the surface, forms furfuraceous scales, and constitutes a new source of irritation. These latter inconveniences are by no means confined to the external organs of generation, as they are usually called, but they extend themselves towards the groins, and to the inside of the thighs; the vestibulum becomes irritated by the discharge, which assumes an ichorous, and shortly afterwards a purulent, appearance. But the quantity of the discharge is by no means comparable with that which is met with in some other diseases of those organs.

" The pus discharged in these cases has usually a fetid odour, the reason of which has been before given. The quantity of the discharge sometimes greatly diminishes, although the disease advances, in consequence of the diminished quantity of blood in the circulation; streaks of blood are occasionally mixed with the pus which escapes, or small coagula come away; or he̍morrhage may take place in quantity sufficient to cause syncope, and to excite great alarm in the mind of the patient.

" If the bladder and the rectum have not sympathized with the disease in its early stage, they will seldom escape at this period, not only from the consent which obtains between all these organs, but also from the disease extending itself to these in common with all the adjacent parts. Such a degree of thickening of the meatus urinarius sometimes takes place, as to impede the passage of the urine, and to require the use of the catheter; shortly after which the urine will gradually and spontaneously escape, not through the urinary passage, but from a communication formed between the posterior part of the neck of the bladder and the vagina. Portions of coagulating lymph will frequently be washed away from the parts in the passage of the urine.

" When the vicinity of the rectum to the uterus is considered, it will be expected that a communication between the former part and the vagina would at the same time take place; but this is not so, as in many cases, where there is a fistula of the bladder, there will be found no communication between the intestine and the vagina.

" Still, however, this circumstance is now and then met with; and, from the moment that it is established, no feces pass through the anus, the external parts forming the channel through which urine from the bladder, feces from the rectum, and pus from the ulceration, are discharged. The stench now becomes intolerable, and the hips of the patient lying almost always immersed in the excreted matters, the soft parts inflame, and sloughing takes place."

In addition to these local symptoms, the constitution suffers; the patient becomes emaciated; the strength diminishes; the functions of the stomach fail; little food is received, and that little is badly digested, and vomitings take place; ill-conditioned sores arise in the angles of the mouth, with apthæ, not only in the mouth, but even in the anus and vestibulum; the tongue becomes sore, pale, dry, and glossy, and sometimes taking a dark-red colour.

" A burning heat of the stomach, and of the intestinal canal, becomes another cause of distress to the patient: this sensation may be dependent, in part, upon the irritating quality of the fluids of the stomach, and in part upon the presence of aphthæ in the primæ viæ. Another symptom, by which the patient is much distressed, is an insatiable thirst, which nothing can allay. This greediness of liquids is not present during the whole of the disease, but it arises at that part of the complaint at which so little blood remains in the blood-vessels as not to furnish fluids for secretions. Something like this takes place in hemorrhages, when they become profuse. To sum up the patient's misery, to render her situation more distressing, she is tortured by a violent, acute, and darting pain, sometimes resembling a sharp instrument piercing the pelvis in different parts; becoming sometimes so intense as to cause the most patient woman to exclaim, and depriving her of all quiet by day, and all repose by night.

"Thus it appears that the situation of a woman labouring under ulcerated carcinoma of the uterus is infinitely more pitiable than that of a woman who has the disease in her breast; for not only are the symptoms more numerous and more insufferable, but she has not the good fortune to be cut off in the progress of the disease by accidental symptoms. In ulcerated carcinoma of the breast, the patient is usually destroyed by hydrothorax; but no such blessing is afforded to the subject of this disease in the uterus, the sufferer being compelled to endure till her frame is exhausted by pain, by vomiting, by want of sleep, by discharge, by an offensive atmosphere, or by gangrene of the integuments."

The reader will perceive, from the preceding account, which contains the substance of Mr. Clarke's work, that nothing has been added to the stock of our knowledge, either as to the diagnosis, prognosis, or pathology of the diseases which it professes to describe. The remaining part relates to the treatment of corroding or carcinomatous ulcer of the uterus, or carcinomatous ulceration of the rectum. These are separately considered by the author; but, as the treatment of all possesses some features in common, we shall combine them, for the sake of brevity.

Inflammation of the diseased organs is the prime mover of all the mischief: our attention should, therefore, be particularly directed to this, keeping in mind that the constitutions we have to deal with cannot bear the vigorous depletion which would be proper in some inflammatory affections, suddenly occurring in persons of firm stamina. Bleeding may be proper, but whether local or general must depend on circumstances, which may vary in every case. It has been remarked by the author, that when a profuse spontaneous bleeding has arisen from some vessel exposed by the ulceration, to the extent of producing syncope, the progress of the symptoms has been thereby arrested. This "arrest of progress," in a former part of the volume, is singularly accounted for, by the mere debility of the patient. "Spontaneous bleedings from the ulcerated surface, producing more sudden debility, will have the same effect in retarding the progress of the disorder:" but this benefit is obviously referrible to the bleeding, as a remedy for inflammatory action.

The following are among the particulars on this head. If the practitioner should find the circulation becoming accelerated, "the skin more than usually hot, flabbiness of the integuments, softness and shrinking of the muscles in different parts of the body, he may presume that some important change has taken place in the diseased organ. If, together with these symptoms, the lancinating pain has been rendered more acute; if the sympathies between the uterus and the adjacent parts, or between that organ and the stomach, have been more than usually called forth; or if, lastly, the mucous discharge has assumed a puriform character, there can be little doubt that a breach of surface has taken place, and that the complaint has acquired its most frightful and distressing character. Sometimes considerable itching ushers in this change: from this point of time, all those cautions which were offered to the patient in the earlier stages of the disease should be insisted upon, as circumstances essential to her future comfort, and necessary to her preservation. If the patient should possess a vigorous constitution, and if she has been in the habit of living freely, the pulse being full, and strong, and hard, it will scarcely be justifiable to omit general blood-letting. Such a practice is, however, seldom requisite; and the propriety of adopting it must be left to the judgment of the practitioner, who must take into consideration this circumstance, that the disease does not exist in an organ essential to life, and *that time will be allowed him to repeat local blood-lettings*, if he should think them to be more proper. So that the treatment should not only be adapted to the symptoms themselves, but should have relation to the organ involved in the disease."

The latter parts of this paragraph appear to us to be sheer nonsense. What would the author think of a physician who should say to his patient, "Your complaint, madam, will unquestionably have a fatal termination; the symptoms will daily increase; but you will not die these six months. I think it fortunate, however, that no *vital* organ is affected; so that we have *plenty of time* for our local bleedings: I shall, therefore, not begin the treatment for the next three months?" Yet this is almost the import of Mr. Clarke's own words. If bleeding be desirable, it cannot be performed at a more seasonable time than before the disease has made much progress.

It will be obvious that, in pursuance of the same indications, strict attention should be paid to diet. Saline purgatives, also, the author adds, when exhibited in very small doses, "possess not only the power of allaying inflammation, by the watery secretions which they produce from the intestines, but they appear also to possess a specific power of tranquillizing the system, when in a state of disturbance and increased action, even when they produce very little sensible effect. Twenty, thirty, or forty grains of magnesiæ sulphas, or potassæ sulphus, may be exhibited twice in each twenty-four hours; and the beneficial effect of these medicines may be still further increased by combining them with very moderate quantities of extractum conii, or extractum hyoscyami."

The next remedy is the warm hip-bath. Frequent injections of tepid fluid into the vagina have the double effect of soothing the parts, and of keeping them free from a discharge, which, in cancer especially, is not only unpleasant by its fetor, but capable of irritating the vagina and extending the disease.

The other local remedies recommended in the corroding ulcer, are mild astringent fluids in the form of injection. If hemorrhagy should occur, solutions of sulphate of alum in decoction of granate bark, or solutions of sulphate of copper or nitrate of silver, may be proper. In the carcinomatous affection, a strong decoction of carrots, or solutions of conium or hyosciamus, in the proportion of four drachms to a pint of water, is advised Opium also, and extract of poppy, in the proportion of half an ounce of the latter or two drachms of the former, may be dissolved in a pint of fluid. Mucilage of starch or quince-seeds form good vehicles for these applications, in consequence of their adhesive property. These fluids may be thrown into the rectum, when they afford no relief in the vagina.

Mr. Clarke adds, that, when opium is used for this purpose, the practitioner should be careful to watch over its effects, as sometimes unpleasant consequences have arisen from the application of *this drug* to the rectum; such as vomiting, syncope, cold extremities, and irregularity of the circulation. Surely he ought to know that the circumstances related are not peculiar to *this drug*, but may be equally experienced from the injection of other narcotics into the rectum: belladonna, tobacco, conium, hyosciamus, and many others, produce analogous symptoms. Can the author inform us wherein the operation of these drugs differ, whether taken into the stomach, introduced into the rectum, or absorbed into the circulation, or injected into the veins? We would venture to suggest, in no essential respect. If, when introduced into, and retained in, the rectum, they have occasionally produced more violent symptoms than is usually observed from their internal exhibition, it is attributable to the difference of dose, and to nothing else. We cannot, then, for the purpose of accounting for these symptoms, adopt Mr. Clarke's supposition, that "the action of the absorbents of the rectum is, in all probability, increased by the inflammatory process in the vicinity.

Plasters and liniments of opium are recommended as auxiliaries, but to what part is not stated. A very diluted mixture of acetic or nitric acid and water are said to form a very soothing application; but, if made too strong, will become highly irritating. Half an ounce of acetic, or ten drops of nitric, acid are recommended to a pint of water: but, as the strength of these acids vary, the dose must be left to the judgment of the practitioner. A lotion of the liq. plumbi and of alum, in decoction of oak bark, or of zinc in water, with some tincture of kino, may be employed according to circumstances.

The two latter are advised on account of their astringent power, when the discharge has become so profuse as to induce great debility; but, if they have the effect of checking the discharge, why not employ them in an earlier stage; so important as it is to the comfort of the patient, to say nothing of the chance of retarding the debility which it may produce.

The class of internal medicines, of whose employment the author speaks, are the narcotics, hyosciamus, conium, stramonium; which are dismissed in a very summary manner, as calculated only to abate suffering by their sedative effects. If, as the author states, these are wantonly employed, the patient will be exposed to another set of symptoms, arising from disturbed state of the stomach and of the brain; as flatulence, heart-burn, eructations, and delirium.

Of the efficacy of belladonna, in the hands of others, he has heard speak; but never uses it, "having seen a patient nearly destroyed by two small doses." Upon the same principle, we might abandon nine-tenths of the materia medica.

A mixture of opiate confection and spiritus ætheris sulphurici, in peppermint water, given in small doses and at short intervals, are advised, as calculated to relieve, in an expeditious and certain manner, the vomiting, singultus, and eructations, more effectually than any other combinations of medicines.

---

### DIVISION II.

### FOREIGN.

ART. II.—*Memoires Physiologique sur les Maladies Purulentes et Putrides sur la Vaccine, &c.* Par B. GASPARD, D.M.—(Journale de Physiologie.)

ART. III.—*Recherches, Physiologiques et Medicales, sur l'Acetate de Plomb.* Par B. GASPARD, D.M.—(Journale de Physiologie.)

WE have already adverted to these memoirs in our Retrospect, and have suggested the bearings which they have upon the theory of medicine, in opposition to those doctrines which have so long taken possession of our schools. While a doubt can exist that the circulating fluids are susceptible of degeneration, and that such a change can be the cause of corresponding disorder in the functions of life, experiments calculated to throw light upon the subject are of inappreciable value. When the advocates of sympathy are asked to account for the effects of putrid miasma upon the animal economy, or of the vapour of lead, or of noxious food, or of medicinal substances, we are coolly referred to nervous agency, as the only means by which the phenomena can be explained. Yet the same effects can be produced by injecting these substances directly into the veins. We have fever and dysentery from the introduction of putrid matter into the circulation, and symptoms analogous to colica pictonum from the acetate of lead. In like manner, the injection of diseased rye into the veins of dogs produces symptoms not unlike those which are experienced in man from the mixture of poisonous seeds with corn.

These facts have been proved by numerous experiments performed by Dr. GASPARD, and which form the subject of these memoirs.

In the first, two drachms of *fetid pus*, slightly diluted, were injected into the jugular vein of a dog. Two hours afterwards, he voided blackish liquid stools, of extreme fetor, which was followed by his speedy recovery.

On the third day, three drachms were injected, and the dog died. No morbid appearances were observable in this case, on dissection.

In other cases a more putrid pus was chosen, and a larger quantity injected. In addition to an aggravated degree of the above-named symptoms, these unhappy victims of experimental physiology had haggard eyes, exquisite sensibility, subsultus tendinum, convulsive paroxysms, furor, delirium, palpitations of the heart, and violent tenesmus: in some, rigidity of the limbs and tetanic spasm were present. The mucous membranes of the intestines were sometimes inflamed; and in one the left ventricle of the heart was also thickened and inflamed.

Two drachms of pus were introduced into the abdomen, through the serous membrane of the testicle. The dog died in twelve hours. The peritoneum and the mucous membrane of the intestines were inflamed.

In another, it was injected into the left pleura. Pleuritic symptoms were produced, from which the dog seemed to be recovering, but was killed, to afford an opportunity of examining the parts within the chest. The pleuræ were inflamed, and covered with albuminous flakes. An inodorous sero-sanguineous fluid was found in the thorax.

These experiments, which had been published in 1809, are again exhibited, in connexion with others, which we subjoin. It may be proper, first, to observe, that the author considers them to lead to the following conclusions.

Pus, introduced into the blood-vessels in small quantity, can circulate in them without inducing death, provided it be expelled from the system by means of some critical excretion, especially of urine or of fecal matter. Even a small quantity, frequently introduced in succession in the same animal, destroys it.

The fatal termination is accelerated when it is injected into the veins in too strong a dose, and then it causes divers severe phlegmasiæ, peripneumoniæ, carditis, dysentery, &c.

It may be absorbed from the serous membranes and from the cellular tissue: but it may be remarked, that this conclusion is not borne out by the experiments hitherto related. It is, however, supported by others which come hereafter.

It is proved also by the disappearance of subcutaneous abscesses, and by the absorption of the pus in confluent small-pox.

The greater part of the symptoms observed in hectic fever are referrible to the presence of pus in the circulating system.

That pus may be present in the circulating system is evident, not only from the preceding experiments, but from the observations of many pathologists. Kerckringius relates that, in a child, who died of marasmus from an abscess in the neck, the latter had corroded the jugular vein, into which it gradually emptied itself. A quantity of pus also was found in the right cavity of the heart. Hollerius, Sulpius, Cornax, Bonetus, Storc, and Laennec, have found deep and extensive ulcerations subsequent to abscess on the internal surface of this viscus. Meckel has seen the interior of the aorta, from the carotids to the iliacs, filled with ulcerations, which had almost entirely destroyed the internal membrane.

The author had no opportunity of trying the injection of the matter of gonorrhœa, syphilis, cancer, and variola, which he intended to have done; but, in two experiments, vaccine matter, dissolved in water, was injected into the veins. In a third, a thread, fully impregnated with this fluid, was passed through the skin of an ass, after the manner of a seton. No vaccine eruption was formed in either case. One of the dogs, in whom the injection was made in the veins, was slightly indisposed, and vomited frequently.

It was desirable to ascertain whether the action of pus upon the animal economy, thus introduced into the veins, depended on its putrid quality, or otherwise. Experiments were then made with putrid matters from other sources.

*Experiment* 14.—Half an ounce of fetid fluid, produced by the simultaneous putrefaction of beef and blood, were injected. The dog instantly had movements of deglutition and dyspnœa, laid on his side, refusing all food, and had an evacuation both of feces and urine. At the end of an hour, his strength was gone; he had repeated gelatinous and bloody alvine discharges, with tenesmus. The conjunctiva was suffused; he had bilious, gelatinous, and sanguineous vomitings; his belly was shining, and sensible to the touch. In three hours he died.

The lungs were found inflamed in a peculiar manner, or rather gorged with blood; and they were of a violet or blackish colour, with ecchymoses or petechiæ; which existed also in the left ventricle of the heart, in the spleen, mesenteric glands, gall-bladder, and subcutaneous cellular tissue. The peritoneum contained some spoonfuls of red serosity; but the mucous membrane of the digestive canal was principally affected; that of the stomach was lightly affected, the duodenum and rectum were considerably so: they were spotted and of a livid colour, covered with a gelatinous and sanguinolent fluid, like wine dregs or the washings of flesh. Moreover, this inflammation was accompanied by slight thickening of the tissues, and had an hemorrhagic or scorbutic appearance.

The author now tried an injection of putrid vegetable matter.

*Exp.* 16.—Two ounces and a half of thick fetid liquor, produced by the fermentation of cabbage-leaves, were injected into the jugular vein of a large-sized dog. He had movements of deglutition, vomitings, and soon fell into a state of exhaustion. Some hours afterwards, the chest was painful on pressure, and respiration difficult. At the end of nine hours, a very copious, fetid, liquid stool was evacuated, black as soot, like mælanic discharges, formed of excrement and mucus and putrid blood. Soon after a similar one was voided, formed of blood and mucus only. On the following day the debility increased; the dog vacillated in walking, but mostly laid on its side; his pulse was small and febrile, thirst ardent, urine natural and copious. The respiration was free, yet weak. But the most remarkable symptom was palpitation of the heart, returning at intervals with extraordinary noise and force. On the fifth day he died. The conjunctiva, pituitary, and buccal membranes were of a red or violet colour, and lined with a large quantity of thick mucus. The lungs were pitchy, and slightly inflamed. The left ventricle of the heart presented several brown spots, or ecchymoses, penetrating even into its substance; its colour was like the dregs of wine, forming a singular contrast with the natural colour of the right ventricle; but the latter was partly filled by a hard, albumino-fibrinous concretion, which extended ramifications of the same colour and consistence into the pulmonary artery, and into the superior venæ cavæ, azygos, axillary, and even right jugular: this was supposed to have caused the violent palpitations of the heart. The œsophagus and stomach appeared healthy; but the mucous membrane of the intestines, and especially of the duodenum and rectum, were inflamed, principally in longitudinal ridges and in irregular patches, which chequered even the external portion of the intestines. Moreover, this inflammation was without thickening of the tissue, without ulcerations, and had the appearance of ecchymosis. The duodenum contained several open vesicles, from which a quantity of sanious blood was forced by pressing the neighbouring mesenteric vein. The mucous glands of the rectum were enlarged: this intestine contained puriform matter, similar to the last evacuations. The mesenteric glands were inflamed and gorged with blood. The gall-bladder, externally, was chequered by brown and violet spots, filled with black, thick, and ropy bile, like melted pitch.

This and other analogous experiments prove that putrid matter, whether vegetable or animal, has the same action upon the system. It remained, then, to be determined what effect would be produced by their absorption, on being injected into the sub-cutaneous cellular texture.

*Exp.* 18.—Three ounces of water, in which putrid meat had been macerated, was injected into the subcutaneous, inguino-abdominal, cellular texture of a little dog, which caused great pain and agitation. Some hours afterwards, a hard and painful tumor was formed. The belly was sensible to the touch. On the following day, the abdomen was tense, the tumor was painful, shining, emphysematous, and of a gangrenous aspect, along the penis and in the neighbourhood of the anus. The eyes were red, and covered with a purulent secretion. Sixty hours after the experiment, the tumor, which had mortified, burst: black and putrid sloughs came away, which laid bare the penis and abdominal muscles. The eyes were filled with matter. On the following day, some amendment and return of appetite was perceivable; the secretion of the eye-lids had abated; the belly was less painful, and the wound of a healthy colour. In a week after this, the recovery of the dog being complete, with the exception of the wound, which rapidly filled with fine granulations, he was submitted to the following experiment.

*Exp.* 19.—Five ounces of very putrid liquid was injected into the peritoneum, at ten intervals, which produced great pain, agitation, and copious flow of urine. After the experiment, his belly was painful on pressure; he had anorexia, vomitings, alvine excretions, painful tenesmus, and was in a state of exhaustion. The stools were mucous, gelatinous, and dysenteric. The wound produced by the former experiment took on a livid, scorbutic appearance; the least pressure on the belly produced convulsive cries.

A large quantity of inodorous, sanguinolent serosity, of the colour of wine dregs, was found in the abdomen. The peritoneum was inflamed, of a violet red colour, and universally ecchymosed. The inflammation was excessive in the course of the mesenteric vessels, and on the concave curvature of the intestines, with black patches and extravasation of blood. All the mucous surface of the digestive canal, from the cardia to the anus, was highly inflamed, and of a deep-red, violet, and blackish colour, especially at its folds. At the same places the stomach was inflamed. The intestinal tube contained a glutinous mucus, which was not sanguineous. The muscular membrane partook in no degree of the inflammation. The bladder, which was empty, contracted, and inflamed externally, was quite white within; the left cavity of the thorax contained a sanguinolent serosity; the spleen and the lungs were covered with spots and ecchymoses. Finally, the wound, which before the injection was covered with healthy granulations, had a dark, scorbutic, or gangrenous aspect.

Another experiment was performed with similar results.

From the two last it will be obvious that the constitutional effects of these injections were produced by absorption; for, independently of the local affection, we observe puriform secretions of the eye-lids, livid spots in the lungs and spleen, sanguinolent effusions in the thorax, and inflammation of the *mucous membrane* of the bowels. It was particularly observed that the latter did not arise from continuity with the peritoneum, as, in parts where the former was strongly inflamed, the latter was not; neither was the intermediate muscular coat affected. The healthy wound also in the nineteenth Experiment became gangrenous.

The general result of the presence of putrid bodies introduced into the circulatory system, whether by injection into the veins or by absorption from serous surfaces, is inflammation of various internal organs, and hemorrhage from the mucous membrane of the bowels. A moment's reflection will show the great similarity which exists between the symptoms and appearances on dissection of the animals subjected to these experiments, and that which is seen on the examinations of persons who have died of yellow fever and dysentery; which, it should be observed, arise, in like manner, from the operation of putrid miasmata on the animal economy.

As we have felt it necessary to omit some of the experiments, it may be proper, in this view of the subject, briefly to recapitulate the accounts of particular symptoms and morbid appearances. It will be readily conceived that the differences which are perceptible in them may be owing to variations in the relative dose of putrid matter injected.

*Injections of Pus into the Veins.*

In the first dog, blackish, liquid, and very offensive evacuations took place from the bowels, with relief of the symptoms, followed by recovery.

*Exp.* 2, on the same dog as the preceding: vomiting, liquid stools, whitish, very fetid, and frequent. She died, but no morbid appearances were found.

*Exp.* 3d and 4th, both experiments on the same dog: lungs inflamed, hepatised, and sunk in water.

*Exp.* 5th.—Vomiting, violent dysenteric tenesmus, liquid and very offensive evacuations, tormina; death in five hours. Dissection: intestines thickened externally, mucous membrane inflamed and swollen.

*Exp* 6.—Terrible nervous symptoms, vision wandering, exquisite sensibility, involuntary startings of the whole body, convulsions, hiccup, dyspnœa, palpitation of the heart, &c. Death in two hours, with horrible convulsions, without evacuations. Dissection: venous blood very coagulable, not parting with its serum; fluid in the pericardium; left ventricle of the heart thickened and inflamed; spots on the inner surface, of the colour of wine dregs.

The following are the results of the *injection into the serous membrane of the testicle:*

*Exp.* 7.—Vomitings, peritoneal inflammation. On dissection, the peritoneum was inflamed, with sanguinolent effusion into the abdominal cavity; the mucous membrane of the intestines was red and inflamed.

*Exp.* 8.—Vomiting, fever, and shining painful state of the abdomen. On dissection, the same appearances as in the preceding case, with albuminous flakes adhering to the peritoneum.

*Injection into the Pleura.*

*Exp.* 9.—Painful respiration, with appearance of pleurisy. Twenty hours after, the dog was apparently recovered, but was killed for the morbid appearances. The two pleuræ were inflamed, and covered with albuminous flakes.

*Injection into the Subcutaneous Cellular Membrane.*

*Exp.* 10.—The pus was not visibly absorbed, but caused suppuration.

*Injection of Putrid Animal Matter into the Veins.*

*Exp.* 14.—Before related, at full length.

*Exp.* 15.—Liquid, bloody, sanious, fetid stools; dysenteric tenesmus. On dissection, same appearances as in the last; mucous membrane was equally inflamed, livid, and of a scorbutic, inflammatory, hemorrhagic aspect.

*Injection of Putrid Vegetable Matter into the Veins.*

*Exp.* 16.—Before related.

*Exp.* 17.—Dysenteric evacuations, bilious vomitings, pain of the abdomen on pressure, and afterwards sanguineous, fetid, black, melænic stools; death in eleven hours. On dissection, the lungs were found gorged and pitchy, without ecchymoses or petechiæ; ecchymoses in the gall-bladder and mesenteric glands.

*Injection into the Cellular Membrane.*

*Exp.* 18.—Before related.

*Injection into the Peritoneum.*

*Exp.* 19.—Before related.

*Exp.* 20.—Matter less putrid, and injected in smaller doses. Acute pain in the abdomen, which was very sensible to the touch; refusal of food; laying on his side; fever, without vomiting or alvine dejections; death in ten hours. Dissection: a large quantity of sanguineous fluid was found in the abdominal cavity. The peritoneum, mesentery, and epiploon, were excessively inflamed, of a violet colour, with spots or dark ecchymoses; adhesions upon different viscera; the bladder was of a red colour externally, but very white within; the stomach was inflamed without, and very healthy within, but the intestines, inflamed externally, *were still more so on their mucous surfaces,* from the pylorus down to the anus, even *in parts which externally preserved their natural colour,* having been protected from the local action of the irritating material in consequence of being buried under other organs. This membrane (the mucous) was thickened, and of the colour of wine-lees.

M. Gaspard then reasons on the possibility of the effects on the animal economy being produced by some one or more of the component parts of putrid matter, as ammonia, hydrogen, carbonic acid, and sulphur: experiments were therefore performed with a view to determine this question.

*Liquid Carbonic Acid injected into the Veins.*

*Exp.* 21.—An ounce and a half of boiled water, strongly shaken up for several days with an equal quantity of carbonic acid gas, was injected into the jugular vein of a dog; a few minutes after he had vomitings, two evacuations, was drowsy, and almost in a state of intoxication, with plaintive breathing. Five hours after the injection, the dog recovered.

Other experiments were made, with no more result.

It is curious that, although liquid carbonic acid did not cause death when introduced into the veins, yet the animal died quickly when it was introduced into the cellular membrane.

*Carbonic Acid injected into the Cellular Membrane.*

Two drachms were injected into the cellular membrane of the neck of a young fox, the subject of a preceding experiment. After some time, he became indisposed and drowsy, and an œdematous tumor formed under the jaw. On the following day the swelling enlarged, and the animal died forty hours after the injection, without any precursory symptoms but progressively increasing debility. On dissection, no appearances were found in the head, chest, or belly; but the cellular membrane of the neck, shoulder, jaw, and cheeks, were lightly inflamed, of a violet colour, and gorged with reddish serosity. The muscles in these parts were of a livid and blackish colour, and affected with a particular inflammation.

Another experiment was followed by the same results.

*Sulphuretted Hydrogen Gas injected into the Veins,*

In Experiments 25 and 26, with very little result: the animal, in one, refused food, was slightly drowsy, &c. but soon recovered.

*Ammonia in Solution injected into the Veins.*

*Exp.* 27.—Twenty drops of weak liq. ammoniæ, with an ounce of distilled water, were injected into the jugular vein of a sucking pig. The symptoms were—evacuation of urine and feces, fever with shivering, belly painful on pressure. Four hours and a half after the injection, he had a liquid brown stool, mixed with clots of blood. He grew better, but continued a week in a languid state, and died nine days after. On the opening of the body, ecchymoses, or numerous petechiæ, were seen in the heart, the intestines, and integuments of the belly. The intestinal appendiculum was very red, swoln, and inflamed in its muscular and serous membranes, with apparent suppuration in the interior.

The same experiment was performed, three different times, on a dog, with similar symptoms and recovery. On one of these occasions, the dog, at the time of the experiment, was almost killed by a species of syncope or suffocation. After the third experiment, the dog was recovering, but was killed by one of a *different kind* almost instantaneously. The left lung was inflamed, but the digestive tube was sound, excepting a large inflammatory spot in the mucous membrane of the duodenum.

*Ammonia injected into the Cellular Membrane.*

*Exp.* 29.—Thirty drops, diluted with three drachms of water, were injected into the cellular membrane of the back of a dog, already emphysematous. Symptoms: liquid, gelatinous stools, acute pain, exquisite sensibility of the skin on pressure. Death in twenty-four hours. Dissection: skin and cellular membrane of the back and sides were largely inflamed, red, violet coloured, and filled with sanguinolent serosity; strong inflammation of the mucous membrane of the duodenum, and of a certain extent of the small intestines.

*Solution of Muriate of Ammonia in the Cellular Membrane.*

*Exp.* 30.—Two drachms of a saturated solution were injected into the cellular membrane. It did not appear to be absorbed, but caused a large inflammatory tumor, which suppurated a long time.

In an experiment, however, performed by another person, one or two drachms of muriate of ammonia, injected into the cellular membrane, caused death in thirty-six hours, with gangrenous inflammation of the mucous membrane of the stomach, spots of blood in the tissue of the heart, and presence of blackish, fetid liquid in the intestines.

Our readers will judge for themselves, from the preceding experiments, whether the effects of putrid substances on the animal economy arise from the ammonia which they contain. The author is of a contrary opinion, because the hemorrhagic inflammation of the mucous membrane of the bowels, observed in the former experiments, did not take place in the latter. It appears to us that the experiments were not made to a sufficient extent to warrant this conclusion. The continued use of alkaline substances, taken internally, are said by HUXHAM to produce "hectical heats, a hot scurvy, nasal and intestinal hemorrhagies, and dysentery."

To prove that the effects of the preceding experiments would not be obtained indiscriminately from other animal fluids, which were not putrid, experiments were made by injecting human semen, saliva, urine, bile, serum effused in dropsy: all the animals, except the one in whom the bile was used, were slightly affected, but soon recovered.

[To be continued.]

---

ART. IV.—*Commentario Clinico per la Cura dell' Idrofobia.* Del Cavaliere VALERIANO LUIGHI BRERA.—(Memoria della Società Italiana delle Scienze residente in Modena. Tom. xviii. parte fisica.)

A MORE awful visitation upon the human race cannot exist than that which forms the subject of this memoir. Hydrophobia spares neither age nor sex: the bloom of youth and the vigour of manhood are alike impotent to avert its destructive career, and the unfortunate victim is rapidly cut off, with an intensity of suffering scarcely to be equalled. Hence we seize every opportunity of placing before our readers any fact, or even speculation, which may tend, in the most remote degree, to throw light upon the disorder, or to excite a spirit of inquiry respecting it. Thus it is that scarcely a month passes but we record something on the subject, however sceptical we may be with regard to the truth of a statement, or the efficacy of the treatment which may be advised. Like the drowning man, indeed, we catch at a straw, when we recount the prophylactics and specifics which appear from time to time; but the cases of this author come before us in a shape, and with an evidence, upon which credulity would be folly; and we press them the more particularly upon public notice, as containing the details of well-conducted, and apparently successful, experiments for securing the system against the ravages of the disease under consideration.

Of thirteen persons bitten by a rabid wolf, on the 1st of November, nine died and four escaped: the latter *only* were submitted to the experiment, which seems to have been successful; while five of those who died were subjected to prophylactic treatment of a different kind. The facts we shall leave to speak for themselves, merely premising that, in three of the successful cases, some horror of water is described to have been experienced.

Girolamo Peverari received two wounds in the neck, which were healed on the 10th. On the 19th, the cicatrices became red and painful. A warm bath was prescribed, with a drachm of mercurial liniment daily, and one of the following pills were directed every three hours:

℞. Oxymur. Hydrarg. gr. ss.
Radicis Belladonnæ pulv. gr. x.
Mellis, gr. vj. Ft. massa in pil. iv. divid.

These were used till the 25th, the quantity of mercury being increased to two grains, and the belladonna to thirty, in each mass of pills. Salivation and inflammation of the fauces having now taken place, the mercury was wholly laid aside, and he continued the bath and the belladonna; the latter of which he took in the dose of four scruples a-day on the 1st of December, and which, by the 19th, was increased to three drachms. On the 1st of December the mercurial friction was resumed, and continued till the 4th of January, when this treatment was discontinued.

In the space of forty-seven days, seven ounces and a half of belladonna, five grains and a half of sublimate, and four ounces of mercurial liniment, had been consumed; and he had used the warm bath fourteen times.

On the second day of this treatment, Peverari perspired greatly, and voided considerable quantities of urine.

Independently of the febrile irritation produced by the mercury, the belladonna occasioned vertigo, vomiting, tremors of the limbs, obscurity of vision, and pains in the eye-balls, which rendered a suspension or diminution of the dose necessary.

*The sight of the bath occasioned, in this man, great anxiety and sense of constriction about the fauces.*

Giroletti Agostino had two wounds in the neck, and others in the arm: the former were healed on the 4th, the latter on the 9th. He had an obtuse pain, with a want of power, in the arm; and for a long time was unable to use the three first fingers of the left hand. On the 19th, the warm bath and mercurial liniment were ordered, with two of the following pills every two or three hours:

℞. Folior Bellad. pulv. gr. xvj.
Hyd. Submur. gr. iv. Mellis q. s. ft. mass. pro pil. xvj.

On the 20th of November, the pain and want of power in the arm were lessened. By the 31st, the dose of the belladonna had been increased to thirty-six grains; but it produced such powerful effects, that it was suspended on the following day. On the 26th, salivation was complete. The belladonna was resumed on this day; sixty grains were given, with four grains of opium.

On the 27th, it was given alone, in the dose of four scruples daily. Urine began to flow copiously, and he had profuse sweats at night.

29*th.*—He had vertigo, obscurity of vision, pain in the eyes, and dryness of the fauces: the belladonna was reduced to a drachm.

*Dec.* 3.—The mercurial frictions were resumed, and continued till the 7th, when salivation was renewed, which lasted through the remaining treatment.

13*th.*—The daily quantity of belladonna was doubled, and on the 19th increased to three drachms. On the 4th of January, treatment was discontinued.

In the space of forty-days, ten drachms of mercurial liniment had been used; ten grains of calomel, and seven ounces and a half of belladonna, were taken internally. He went nine times in the bath.

Besides the usual effects of the belladonna, vertigo, obscurity of vision, pain in the eyes, acidity of the fauces, and tremors of the extremities, this remedy induced a general debility, which was relieved by combining opium with it: the latter, however, produced suppression of urine. *The warm bath could not be long used, as the sight of water produced great oppression about the præcordia, and sense of suffocation as soon as he entered it, so that he could not remain longer than eight or ten minutes.* He experienced also a degree of repugnance in drinking water for several days: nevertheless, he could take wine with ease and pleasure.

Giroletti Agostino. His wounds were healed on the 7th. On the 19th, six grains of belladonna were prescribed four times a-day, with mercurial liniment, and a warm bath. The belladonna was daily increased to two drachms. Perspiration and copious flow of urine took place on the second day. The treatment ended on the 31st of December.

Six drachms of mercurial liniment were consumed; and upwards of eight ounces of belladonna were taken internally.

He went into the bath ten times, feeling no inconvenience from its use, as in the former case.

Fucchetti Carlo Giuseppe. This man was bitten in the right arm, in the hand, and left hip. The wounds were healed on the 18th of November. His child had just fallen a victim to the disease, and his wife died of it on the 1st of December. Deeply afflicted by the latter of these events, he fell into a profound melancholy. He began with two grains of belladonna every four hours, and a drachm of mercurial liniment was ordered for daily friction. The belladonna was increased gradually to three drachms in twenty-four hours: it produced the usual effect of dryness in the fauces, vertiginous paroxysms, obscurity of vision, temporary blindness, increase of urine, and night sweats. Slight salivation came on about the 26th. The daily use of the warm bath was now added to the other treatment, which was continued until the 5th of January, 1805, making a period of forty-seven days. He had in this time taken eight ounces of belladonna.

In the course of the treatment he had, in addition to the symptoms above named, violent palpitations at night, with cold chills, succeeded by icterical appearances, especially in the eyes. On the 21st of December, he had some symptoms of incipient hydrophobia, and complained of pain in the cicatrices of his thigh; and, although in a state of great emaciation and depression, his eyes became vivid and fierce. *He resolutely refused to go into a warm bath; but, having been forced into it, he was scarcely immersed when he was attacked by anxiety and convulsions to such a degree as to make it necessary to withdraw him immediately.* This symptom took place on two consecutive days, upon which a seton was inserted into the painful part; and it was at this time that the belladonna was pushed to its utmost dose.

[To be continued.]

